# Should patients with obesity be more afraid of COVID‐19?

**DOI:** 10.1111/obr.13083

**Published:** 2020-06-24

**Authors:** Anna Maria Rychter, Agnieszka Zawada, Alicja Ewa Ratajczak, Agnieszka Dobrowolska, Iwona Krela‐Kaźmierczak

**Affiliations:** ^1^ Department of Gastroenterology, Dietetics and Internal Diseases University of Medical Sciences Poznan Poznan Poland

**Keywords:** co‐morbidities, COVID‐19, obesity, risk factors

## Abstract

COVID‐19 crisis has lasted since the late 2019 to the present day. The severity of the disease is positively correlated with several factors, such as age and coexisting diseases. Furthermore, obesity is increasingly considered as a yet another risk factor, particularly, because it has been observed that people suffering from excessive body weight may experience a more severe course of COVID‐19 infection. On the basis of current research, in our nonsystematic review, we have investigated the extent to which obesity can affect the SARS‐CoV‐2 course and identify the potential mechanisms of the disease. We have also described the role of proper nutrition, physical activity and other aspects relevant to the management of obesity.

## 1 INTRODUCTION

The crisis caused by coronavirus disease 2019 (COVID‐19), also known as severe acute respiratory syndrome coronavirus 2 (SARS‐CoV‐2), began in late 2019 in Wuhan (capital of Hubei, China) and has spread worldwide in early 2020.^1^, ^2^ According to World Health Organization (WHO) report of May 16, since the beginning of the outbreak over 4 million cases have been confirmed, more than 300 000 of which have been fatal.^3^

However, we are also facing another pandemic—the global prevalence of obesity almost tripled between 1975 and 2016, and it is currently estimated that over 2 billion people suffer from excessive body weight.^4^, ^5^ The current estimates indicate that obesity rates will continue to rise until at least 2030.^6^ It is generally accepted that obesity is associated with an increased overall mortality, where the life expectancy of an individual suffering from severe obesity is reduced by 5–20 years.^7^ Moreover, it also increases the risk of developing several co‐morbidities, also associated with the severity of the COVID‐19 infection, such as cardiovascular disease (CVD), type 2 diabetes (T2DM), kidney disease or numerous types of cancer.^8^, ^9^ Additionally, obesity also increases the risk of developing pneumonia and other viral respiratory tract infections.^10^, ^11^

Due to the fact that excessive body weight constitutes a serious global issue, it requires even more attention because it is presumably associated with the severity of the current COVID‐19 pandemic. Although the current data are scarce, in our nonsystematic review, we have tried to investigate how many of SARS‐CoV‐2 patients suffer from obesity and to identify the potential mechanisms which would further explain the association between obesity and SARS‐CoV‐2 severity. Moreover, we have also described both nutritional and behavioural aspects which could provide a basis for the potential guidelines.

## 2 HOW MANY COVID‐19 PATIENTS SUFFER FROM OBESITY?

Although the data regarding the impact of SARS‐CoV‐2 in individuals with obesity are limited and their association has not been fully defined yet, it has been observed that people suffering from excessive body weight may experience a more serious COVID‐19 infection.^12^, ^13^ It is particularly evident in individuals who are affected by other risk factors mentioned earlier, which associated with a more severe course of the disease. This association was also observed during influenza A pandemic in 2009, where obesity was recognized as an independent risk factor of complications. In fact, patients with obesity, following a high‐fat diet, suffered over 40% longer than those without excessive body weight.^14^, ^15^ Therefore, it seems that obesity during COVID‐19 outbreak requires more attention, especially in the western countries where the prevalence of obesity is high.^16^

According to the COVID‐19‐Associated Hospitalization Surveillance Network in the United States at the turn of March 2020, 48% of hospitalized patients presented obesity (range across age groups = 41–59%).^17^ Among patients admitted to the intensive care unit in a French hospital, 47.6% and 28.2% had BMI > 30 kg/m^2^ and BMI > 35 kg/m^2^, respectively, and obesity constituted a risk factor for COVID‐19 severity.^18^ According to the ICNARC (Intensive Care Unit National Audit and Research Centre) report, over 38% of patients at the clinical care unit suffered from obesity, and patients with BMI > 30 died in the critical care units in 57.6% of the cases.^19^ Furthermore, in the study by Petrilli et al^20^ including over 4000 cases, obesity was the strongest predictor of critical illness, substantially higher than pulmonary, or cardiovascular diseases. Similar results were observed in other studies where BMI values were significantly increased in severe cases and non‐survivors.^21^, ^22^, ^23^, ^24^, ^25^ Additionally, severe obesity (≥35 kg/m^2^) was also associated with an increased risk of ICU (intensive care unit) admission among hospitalized patients admitted in the United States.^26^ A summary of obesity prevalence in various research studies and different populations is presented in Table 1.

**TABLE 1:** BMI values and obesity prevalence among COVID‐19 patients

Data/Location	Bodyweight Statistics			
COVID‐NET (14 states), March 1–30, 2020 17	Obesity prevalence—% of total cases			
	Overall	18–49 years	50–64 years	≥65 years
	48.3%	59%	49%	41%
Four hospitals in the USA February 17–April 5, 2020 26	ICU admitted		Non‐ICU admitted	
	56.8%		39%	
20	Not hospitalized		Hospitalized	
	14.4%		39.8%	
Union Hospital in Wuhan January 20–February 15, 2020 22	BMI values among different groups			
	General	Critical	Survivors	Nonsurvivors
	20.0	25.50	>25 among 18.95% of overall	>25 among 88.24% of overall
Jianghan University Hospital January 3–January 11, 2020 21	General		Critical	
	22.0		27.00	
Taizhou Public Medical Center January 1–‐March 11, 2020 25	Nonseverely ill		Severely ill	
	23.20		24.78	
Three hospitals in Wenzhou January 1–February 29, 2020 23	Severe cases among healthy weight patients (9.5%)		Severe cases among patients with obesity (37.8%)	
	22.7		28.3	
Roger Salengro Hospital February 27–April 5, 2020 18	No invasive mechanical ventilation		Invasive mechanical ventilation	
	27.0		31.1	

## 3 HOW COULD OBESITY AFFECT THE COURSE OF COVID‐19?

The receptor for COVID‐19, similarly to other coronaviruses, has a high affinity for human angiotensin‐converting enzyme‐2 (ACE‐2), expressed in type 1 and 2 alveolar epithelial cell and endothelium, but also in such organs as the heart, endothelium, pancreas and the intestinal epithelium.^27^ In fact, ACE‐2 is considered to be the receptor allowing the entry of COVID‐19 into the host cells by means of the activation of the renin‐angiotensin‐system (RAS).^28^ SARS‐CoV‐2 affinity for the ACE‐2 receptor is higher than in the case of SARS‐CoV.^29^ It is vital to point out that the adipose tissue might be prone to SARS‐CoV‐2 because the expression of ACE‐2 is higher in adipose tissue than in the lung tissue.^15^ Nevertheless, there is no current evidence for direct COVID‐19 infection of adipose tissue.^26^, ^30^, ^31^ At this point, it should be emphasized that the existing recommendations do not advise a discontinuation of ACE inhibitors and angiotensin receptor blockers, used nowadays as a treatment for hypertension unless the physician recommends otherwise.^32^, ^33^ ‘Cytokine storm', that is, one of the mechanisms responsible for the severity of COVID‐19, is the hyperactivation of the immune system and is associated with an increased level of interleukin (IL)‐6, interferon γ and other pro‐inflammatory cytokines.^34^, ^35^, ^36^ Taking the abovementioned facts into account, the adipose tissue synthesizes several proinflammatory adipokines and cytokines which can weaken the immune response and, thus, could constitute the link between obesity and the severity of COVID‐19.^18^, ^34^, ^37^ Additionally, several adipokines secreted by the adipose tissue are associated, mainly negatively, with pulmonary function.^38^, ^39^ Leptin is involved in the surfactant production and the development of lungs in the neonatal period, but it also participates in the ventilatory drive regulation.^40^ Another adipokine‐omentin may play a role in the pathogenesis of asthma; however, it can also have a protective effect on the pulmonary endothelial function and decrease pulmonary permeability and inflammation.^41^ Reduced omentin, ghrelin and adiponectin levels were observed in individuals suffering from obstructive sleep apnea (OSA).^41^, ^42^

Obesity can also predispose to a greater viral shedding, leading to a greater viral exposure.^37^ Because obesity‐related co‐morbidities are commonly identified among COVID‐19 individuals, they can also account for additional risk factors regarding the severity of COVID‐19 complications in obesity.^34^, ^43^, ^44^ Obesity predisposes to several co‐morbidities and mechanisms which lead to a more severe course of COVID 19.^10^ Although, these co‐morbidities were mentioned before, it is crucial to stress that metabolic disorders, such as hypertension, insulin resistance, dyslipidemia or prediabetes, which frequently occur in patients suffering from obesity, also predispose to a poorer COVID‐19 outcome.^8^, ^9^, ^43^

Furthermore, research studies indicate that individuals suffering from obesity present lower vitamin D levels.^45^, ^46^, ^47^, ^48^ In fact, vitamin D could also reduce the cytokine storm—there is also a possible inverse association between the vitamin D level and inflammatory biomarkers, such as IL‐ 6, IL‐8, and monocyte chemoattractant protein 1 (MCP‐1),^46^ although this association has not been entirely confirmed. Moreover, vitamin D could also induce cathelicidins and defensins which can lower the viral replication rate.^49^ It remains to be seen if a deficient, or insufficient vitamin D level could constitute another risk factor for COVID‐19 severity. On the other hand, vitamin D can modulate the expression of ACE‐1 and ACE‐2 and have a protective effect on lipopolysaccharide‐induced lung damage.^50^

The characteristics of patients with obesity regarding possible factors affecting a more severe COVID‐19 course are shown in Table 2.

**TABLE 2:** The characteristic of patients with obesity‐associated with high risk and worse outcome of COVID‐19

Pulmonary function	Pulmonary oedema, lung damage, increased pulmonary vascular permeability, impaired gas exchange, reduced oxygen saturation of blood, decreased ERV and FRC, lower muscle strength, lower lung volume
Co‐morbidities	Atherosclerosis, cancer, epicardial adipose tissue inflammation, asthma, ARDS, OSAS, COPD, T2DM, CVD
Metabolic abnormalities	Hypertension, insulin resistance, prediabetes, dyslipidemia
Adipokines and cytokines	↑ TNF‐α, ↑ IL‐6, ↑ IL‐8, ↑ CRP, ↑ MCP‐1, ↑ leptin, ↓ adiponectin, ↓ omentin,
Vitamin D status	Usually deficient or insufficient level

Abbreviations: ERV, expiratory reserve volume; FRC, functional residual capacity; ARDS, acute respiratory distress syndrome; T2DM, type 2 diabetes mellitus; CVD, cardiovascular disease; OSAS, obstructive sleep apneas; COPD, chronic obstructive pulmonary disease; TNF‐α, tumour necrosis factor α; IL‐6, interleukin 6; CRP, C‐reactive protein; MCP‐1, monocyte chemoattractant protein 1; ↑, higher among patients suffering from obesity; ↓, lower among patients suffering from obesity.

## 4 OBESITY, RESPIRATORY DYSFUNCTIONS AND COVID‐19

Most of the COVID‐19 patients present symptoms of a respiratory disease.^51^, ^52^ Hospitalization rates and the number of respiratory infections are higher in individuals with obesity than in normal‐weight patients.^40^ There are several respiratory dysfunctions and impaired respiratory mechanisms associated with obesity which can deteriorate the course of SARS‐CoV‐2.^53^, ^54^, ^55^ First of all, patients with obesity have an increased airway exchange and impaired gas exchange.^24^, ^54^ An excessive adipose tissue amount in the abdominal area can decrease diaphragmatic excursion and impair ventilation of the base of the lung, which in turn can lead to the reduced blood oxygen saturation.^18^ Additionally, both lung volume and muscle strength are lower among patients with obesity. In the study conducted by Jones et al,^56^ an increased BMI exponentially decreased the functional residual capacity (FRC) and expiratory reserve volume (ERV) among patients with obesity and were equal to 47% and 75% of the values for lean individuals, respectively. Vital capacity and total lung capacity can also be decreased among patients with extreme obesity.^57^ Secondly, obesity is linked to asthma, chronic obstructive pulmonary disease (COPD), obesity hypoventilation syndrome (OHS), pulmonary hypertension and OSA.^40^, ^53^ Moreover, excessive body weight also impairs the course of acute respiratory distress syndrome (ARDS) and COPD, as well as impacts pulmonary function during acute episodes.^58^, ^59^ Obesity also increases the risk of hypoventilation syndrome in the ICU individuals and can lead to a respiratory failure when ARDS is present.^30^, ^35^, ^60^

The likely mechanisms which emphasize these effects are poorly understood, but it is suggested that obesity‐induced imbalances in adipokine levels could impair pulmonary vascular endothelial function and cause lung damage.^61^ Furthermore, not only has leptin been involved in the physiological but also in the pathological conditions of the respiratory system, such as COPD, OSA and asthma.^62^ In fact, due to high leptin levels among patients with obesity, leptin resistance can be the result of upregulation of SOCS‐3, which also negatively regulates antiviral interferons (INFs) signalling.^63^ Furthermore, IFN deficiency might constitute the link between the risk factors, such as obesity, and the severity of pulmonary disorders.^62^

## 5 OBESITY PARADOX AND COVID‐19

Several studies demonstrated that individuals with excessive body weight had improved survival rates, or a chronic disease prognosis, for example, of CVD when compared with healthy body weight individuals.^64^, ^65^, ^66^, ^67^ This phenomenon was called ‘the obesity paradox' and, according to CVD disease, it may relate to even 30% of patients with obesity.^68^ Whether the obesity paradox will be present among COVID‐19 patients remains to be seen, nevertheless, the phenomenon was reported among other respiratory diseases, such as COPD or ARDS.^53^, ^69^ Its pathophysiological basis remains unknown; however, an increased BMI seems to be associated with a better survival and a slower decline in the lung function in patients with a mild course of chronic obstructive pulmonary disease.^70^ It may be partially explained by the fact that a severe form of COPD can lead to weight loss and later to cachexia—it would seem that obesity would prevent this.^71^ Moreover, the obesity paradox in COPD could also be explained by adipokines, because certain individuals can have a more ‘favourable' adipokine profile.^71^ Regardless of the obesity paradox in COPD, a low‐energy diet which leads to weight loss, combined with physical activity, is still recommended among patients with obesity and respiratory disorders.^72^ It alleviates the symptoms and improves the functional capacity and strength, as well as helps to maintain a free‐fat mass.

## 6 WHAT GUIDELINES SHOULD WE FOLLOW?

To the best of our knowledge, there are no specific guidelines concerning the nutritional treatment of COVID‐19 patients with obesity. However, such organizations as WHO, or The European Association for the Study of Obesity (EASO) have prepared nutritional guidelines and tips regarding nutrition during quarantine.^73^, ^74^, ^75^, ^76^ It seems obvious that because obesity possibly is associated with the severity of COVID‐19, it is recommended to apply a hypocaloric diet to reduce body weight.^31^ What is more, weight loss is also beneficial in terms of lung function.^77^ Several behavioural and nutritional aspects could be essential among patients with obesity, and they should be considered when preparing future guidelines.

On the basis of the existing knowledge, an adequate intake of vitamins A, E, B3, B6, B12, D, and folate, as well as zinc, copper, selenium, iron, and omega‐3 polyunsaturated fatty acids is essential for the maintenance of proper immune function.^36^, ^75^, ^78^, ^79^, ^80^ Although micronutrient insufficiency and nutritional status increase the risk of COVID‐19 infection and could worsen its outcome, the use of supraphysiologic amounts to improve the course of COVID‐19 is not currently recommended.^81^, ^82^ However, studies have demonstrated that the intake of several vitamins and micronutrients, such as vitamin A, C, B1, potassium, magnesium, calcium, or iron is usually insufficient in patients suffering from obesity and therefore should be taken into account.^83^ Whether vitamin D supplementation should be increased in COVID‐19 period remains unclear and is not currently recommended. In fact, more randomized‐controlled trials should be performed to confirm these suggestions.^84^

It has been shown that a high consumption of saturated fatty acids (SFAs) can induce lipotoxic state, cause adipose tissue inflammation and promote the activation of the innate immune system due to the activation of toll‐like receptor 4 (TRL‐4).^85^, ^86^ In the animal model, a high‐fat diet induced MCP‐1 for macrophage infiltration into the lung tissue and an increased lipopolysaccharide induced interleukin‐1β and tumour necrosis factor α (TNFα) production.^87^ A high intake of both protein and fat may be associated with higher levels of plasma interleukin‐6 and TNFα.^88^ However, the interaction between nutrition and immunology is extensive and very complex; thus, more randomized‐controlled trials should be conducted in this area.^89^

Psychosocial burdens, for example, stress or depression, have been associated with obesity, and a psychological support is an essential approach to managing obesity.^90^ During COVID‐19 pandemic, increased psychological distress was observed both in infected individuals and in the general population.^91^ Additionally, social isolation was found to be a predictor of an increased mortality, as well as a possible cause of depression and anxiety in the youth.^92^, ^93^ It is worth noticing that individuals suffering from obesity are usually more vulnerable to distress and psychological impact of COVID‐19 quarantine can influence the proper obesity management.^94^ Psychological distress can also lead to a binge and emotional eating among bariatric surgery patients (in time before or after the surgery), which can be associated with poorer long‐term results.^94^ However, the association between psychological aspects and obesity during the current epidemiological situation needs to be further investigated; psychological support should definitely constitute one of the elements of the proper obesity management during COVID‐19 pandemic.

Although the recent statistics are limited, it could be assumed that during the prolonged SARS‐CoV‐2 quarantine the level of physical activity is lower than usual. In fact, physical inactivity not only predisposes to weight gain but can also lead to the loss of strength, skeletal muscle mass and immune competence.^82^ It is vital to bear in mind that quarantine reduced the physical activity level not only for habitually, physically active individuals but also for those whose activity was associated with work, or school, as well as those who mainly work physically.^93^ Physical activity has a positive effect on lung function; it lowers the risk of respiratory infections and may partially counteract binding of COVID‐19 to the ACE‐2 receptor.^95^, ^96^ Therefore, moderate to vigorous physical activity, but not of high intensity, should be recommended as a supporting, preventive component, particularly for patients with obesity among whom additional weight gain can lead to serious health consequences, including rapid loss of FRC and ERV.^56^, ^95^, ^96^

## 7 CONCLUSIONS

- New observational studies indicate that higher BMI values are significantly increased in patients with severe course of the disease and nonsurvivors.
- Considering the existing knowledge, behavioural management of obesity, including weight loss, should be considered as one of the targets for preventing care and viable strategy, especially during COVID‐19 pandemic.
- We strongly advise to perform the measurement of body weight (with the assessment of adipose tissue) in every COVID‐19 patient, because obesity deteriorates the infection course and is associated with an increased mortality among COVID‐19 patients. Additionally, guidelines on nutritional and behavioural support among COVID‐19 patients with obesity are necessary.
- At this point, it is not known what kind of chronic medical conditions, and if any, will be seen in COVID‐19 recovery individuals. It could be suggested that quite a few of them may need a special dietary treatment and behavioural interventions. Moreover, post ventilation‐acquired dysphagia in SARS‐CoV‐2 survivors could be a challenge and will require a special nutritional support.

## CONFLICT OF INTEREST

The authors declare no conflict of interest.
